# Assessment of seasonal forecasting errors of the ECMWF system in the eastern Indian Ocean

**DOI:** 10.1007/s00382-023-06985-3

**Published:** 2023-10-12

**Authors:** Michael Mayer, Magdalena Alonso Balmaseda, Stephanie Johnson, Frederic Vitart

**Affiliations:** 1 Research Department, European Centre for Medium-Range Weather Forecasts, Reading, UK; 2 Department of Meteorology and Geophysics, University of Vienna, Vienna, Austria

**Keywords:** Seasonal forecasting, Indian Ocean, Climate variability, Coupled modelling

## Abstract

The interannual variability of the Equatorial Eastern Indian Ocean (EEIO) is highly relevant for the climate anomalies on adjacent continents and affects global teleconnection patterns. Yet, this is an area where seasonal forecasting systems exhibit large errors. Here we investigate the reasons for these errors in the ECMWF seasonal forecasting system SEAS5 using tailored diagnostics and a series of numerical experiments.

Results indicate that there are two fundamental and independent sources of forecast errors in the EEIO. The first one is of atmospheric nature and is largely related with too strong and stable easterly atmospheric circulation present in the equatorial Indian Ocean. This induces an easterly bias which leaves the coupled model predominantly in a state with a shallow thermocline and cold SSTs in the EEIO. The second error is of oceanic origin, associated with a too shallow thermocline, which enhances the SST errors arising from errors in the wind. Ocean initial conditions, which depend on both the quality of the assimilation and the ocean model, play an important role in this context. Nevertheless, it is found that the version of the ocean model used for the forecast can also play a non-negligible role at the seasonal time scales, by amplifying or damping the subsurface errors in the initial conditions.

Errors in the EEIO are regime-dependent, having different causes in the warm (deep thermocline) regime with strong atmospheric convection and in the cold (shallow thermocline) regime. Errors also exhibit decadal variations, which challenges the calibration methods used in seasonal forecasts.

**Supplementary Information:**

The online version contains supplementary material available at 10.1007/s00382-023-06985-3.

## Introduction

The tropical Indian Ocean exhibits rich climate variability from subseasonal (e.g., the Madden-Julian Oscillation; see Madden and Julian 1971, 1994; Zhang 2005) to seasonal time scales (e.g., the Indian Ocean Dipole, see Saji et al. 1999). Atmosphere-ocean coupling is strong in this region due to strong air-sea fluxes associated with the high sea surface temperatures (SSTs). Thus, Indian Ocean SST variability is associated with pronounced atmospheric variability impacting not only adjacent continents (e.g., the Southeast-Asian monsoon [Ashok et al. 2001], or Australian monsoon, [Heidemann et al. 2023]), but also other tropical ocean basins (e.g., Schott et al. 2009; Wang et al. 2019; Jourdain et al. 2016; Mayer and Balmaseda 2021) and the southern (e.g., Cai et al. 2011; McIntosh and Hendon 2018) and northern (e.g., Garfinkel et al. 2014; Hardiman et al. 2020) extratropics through teleconnections. Thus, the Indian Ocean is a relevant region for seasonal forecasting, as predictive skill in this region can be a source of predictability in remote regions as well.

The skill of dynamic forecasting models in predicting the state of the Indian Ocean Dipole (IOD) is generally lower compared to that of El Nino – Southern Oscillation (ENSO). For example, for the North American multi-model ensemble mean, Wu and Tang (2019) found anomaly correlation coefficients order 0.5 for forecasts of the IOD in boreal fall (May start dates), while L’Heureux et al. (2019) found anomaly correlations larger than 0.7 for forecasts of the Nino 3.4 ENSO index in boreal fall (also May start dates). Prediction skill of Indian Ocean SSTs is similar in other models such as the UK Met Office system and contributes to the rather moderate skill in seasonal prediction of variations of the southeast-Asian monsoon (Johnson et al. 2017; Martin et al. 2021).

Johnson et al. (2019) showed that ECMWF’s currently operational seasonal forecasting system SEAS5 exhibits particularly low skill in the eastern Indian Ocean, where most of the oceanic variability associated with the IOD takes place. In fact, the continuous ranked probability skill score (Wilks 2011) of SEAS5 forecasts in the EEIO is < 0, i.e. climatology provides better forecasts than SEAS5. The EEIO is one of the very few regions globally where predictive skill of SEAS5 was significantly reduced compared to its predecessor SEAS4 (Molteni et al. 2011). The low skill of SEAS5 is associated with overly dispersive forecasts and a cold bias in the eastern equatorial Indian Ocean (EEIO).

**Fig. 1:**
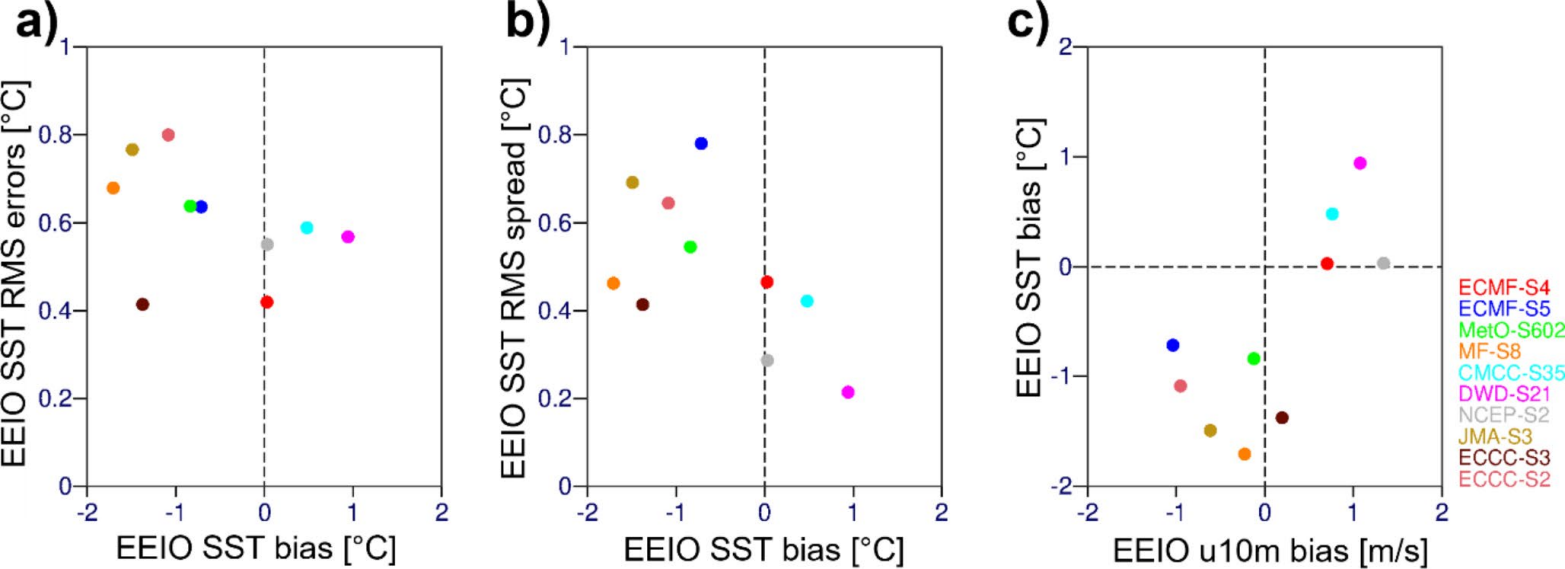
Relationships between Aug-Sep-Oct mean EEIO SST bias, root-mean-square (RMS) error, RMS spread and 10 m zonal wind bias in the C3S multi-system seasonal re-forecasts initialized on 1 May from 1993 to 2015 (**a**) SST RMS errors versus SST bias (**b**) SST RMS spread versus SST bias. (**c**) SST bias versus zonal 10 m wind bias. Models are labelled by an abbreviation of their forecasting centre and their system value in the Copernicus Climate Data Store and include ECMWF’s System 4 (ECMF-S4), ECMWF’s SEAS5 (ECMF-S5), the UK Met Office’s GloSea6 (MetO-S602), Meteo-France’s System 8 (MF-S8), CMCC’s SPS 3.5 (CMCC-S35), DWD’s GCFS2.1 (DWD-S21), NCEP’s CFSv2 (NCEP-S2), JMA’s CPS3 (JMA-S3), ECCC’s GEM5-NEMO (ECCC-S3) and ECCC’s CanCM4i (ECCC-S2). Results are based on ten ensemble members from the 1993–2015 re-forecasts of the ten forecast systems. Some of the forecast centres initialize using lagged ensembles, in that case the 10 members closest to the start date of 1 May were used

We note that Cai and Cowan (2013) and more recently McKenna et al. (2020) found similar problems in a large fraction of free model runs performed within Coupled Model Intercomparison Project Phase Three (CMIP3; Meehl et al. 2007) and CMIP5 (Taylor et al. 2012). Many of the seasonal forecast models provided by the Copernicus Climate Change Service (C3S, 2018) also exhibit a behaviour comparable to SEAS5. Results in Fig. 1 demonstrate that the errors in the EEIO in ECMWF SEAS5 are shared by many other forecast systems. Figure 1a and b compare the climate mean SST bias in the EEIO to the root-mean-square of the error and the root-mean-square spread of the bias-corrected ensemble re-forecasts, respectively. When the SST bias is small, the RMS error is also at a minimum, with the exception of ECCC-S3, which has the lowest RMS error despite a large cold SST bias (Fig. 1a). This figure also shows that the largest SST biases and RMS errors are associated with cold SST biases. The cold SST biases are also associated with larger ensemble spread (Fig. 1b), while warmer SST biases are associated with smaller ensemble spread. This suggests that large, cold SST biases are associated with lower skill and higher uncertainty in the SST forecast and improved understanding of the errors of the ECMWF system in the EEIO is thus of wider relevance. Figure 1c relates mean SST bias and mean zonal wind bias, which will be discussed later.

Here, we present diagnostics of atmosphere and ocean errors that aim at a better understanding of the causes of the biases in the EEIO and what led to their degradation from SEAS4 to SEAS5. Results from various numerical experiments help to disentangle the role of ocean initial conditions, the ocean model, ocean resolution, and the atmospheric model. Besides assessment of forecast bias as a function of start month and lead time, we also address temporal variations of forecast errors across the SEAS5 hindcast period (starting in 1981). The focus of this study are the errors on the seasonal time scale, but Mayer et al. (2022) show that the errors are already present in the first weeks of the forecasts.

The remainder of the paper is organized as follows. Section 2 describes used data and methods, and results are presented in Sect. 3. Section 3.1 provides an overview of forecast biases in SEAS5 and their changes compared to SEAS4. The evolution of surface and sub-surface biases in SEAS5 and several other numerical experiments is explored in Sect. 3.2 and 3.3, respectively. State-dependence of ocean and atmosphere errors is evaluated in Sect. 3.4 and 3.5, respectively. Long-term variations in the SST forecast errors are discussed in Sect. 3.6. The paper concludes with a summary and conclusions in Sect. 4.

## Materials and methods

The starting point for all diagnostics is SEAS5, the currently operational seasonal forecasting system of ECMWF (Johnson et al. 2019). It uses the IFS model at Tco319 horizontal resolution on L91 vertical hybrid levels coupled to the NEMO3.4 (Madec et al., 2017) ocean model run at ¼° horizontal resolution on 75 levels (ORCA025 Z75). SEAS5 is initialized from ORAS5 (Zuo et al. 2019) in the ocean and up to 2016 from ERA-Interim (Dee et al. 2011) in the atmosphere. From 1st Jan 2017, the atmosphere in SEAS5 has been initialized from operational analyses. SEAS5 hindcasts are available every month back to 1981 (with up to at least 7 months lead time), but here we mostly limit our analyses to the period from 1993 onward, as this is the beginning of the standard period for calibration of the forecasts.

The role of ocean resolution is assessed by exploring results from a version of SEAS5 that uses a lower ocean resolution (ORCA1 Z42) but is otherwise identical to SEAS5, which we term SEAS5_LR. It is initialized from ORAS5_LR, a low-resolution version (ORCA1 Z42) of ORAS5.

The impact of ocean initial conditions in a low-resolution setup is assessed through an experiment using ocean initial conditions from ORAS4 (Balmaseda et al. 2013), the predecessor of ORAS5. We term it SEAS5_LR_O4. ORAS4 is run on ORCA1 Z42 and differs from ORAS5 (and consequently ORAS5_LR) in several aspects, one crucial point being the use of the older version 3.0 of the NEMO ocean model. Please note however that SEAS5_LR_O4 *forecasts* use NEMO version 3.4, i.e., the same as SEAS5_LR.

The relative contributions of atmosphere and ocean models to the diagnosed biases are assessed through two different types of experiments. The contribution of the atmosphere model is addressed through uncoupled atmospheric hindcasts using observed SST from ERA5 (Hersbach et al. 2020), named SEAS5_obsSST. The atmospheric model is otherwise the same as that used for SEAS5. For assessment of the contribution of the ocean model, we use a coupled forecast experiment in which atmospheric winds, temperature, and moisture are relaxed towards ERA5 analyses with different strengths. These experiments are termed FC_AtmNudge_XX, where XX is a placeholder for the relaxation time of the nudging or the associated control (ctrl) run without atmospheric nudging. As a result, the ocean “sees” more realistic winds especially in the experiment with strongest (2-hourly) nudging, but the atmospheric flux feedback that could act on SSTs is inevitably eliminated. Table 1 summarizes the set of hindcast data employed for this study.

The assessed physical quantities are SST and vertically resolved temperature in the ocean, 10 m u-wind (u10m), v- wind, and precipitation in the atmosphere. Validation data for biases in the ocean subsurface are gridded ocean analyses from Hadley EN4 (Good et al. 2013). Although the ocean reanalyses assimilate in-situ observations from the EN4 collection, these observations are ingested differently in the ocean reanalyses (they use variational data assimilation with a dynamical ocean model) and in the model-independent gridded EN4 dataset. Therefore, systematic differences can still tell about drift of the assimilating model. However, since representation of interannual variability in EN4 can be affected by the lack of observations, especially in the pre-Argo era, we validate oceanic variability in the forecasts against their own analyses (e.g., SEAS5 against ORAS5), as the dynamical reanalyses ingest additional observational information (e.g., altimeter data) that helps constraining variability. Atmospheric variables and SSTs (with the exception of Fig. 7) are validated against ERA5. As a reanalysis product, ERA5 may differ from observed products, especially in fields like precipitation., but the focus of this study is to assess model biases due to errors in fields such as SSTs, i.e., ERA5 data represents the precipitation that the forecast model would produce in the absence of bias in all relevant fields determining precipitation. Precipitation biases of ERA5 compared to independent validation data are discussed elsewhere (e.g., Lavers et al. 2021).

The employed diagnostics use the first 10 ensemble members unless otherwise stated. The focus region of this study is the eastern equatorial Indian Ocean (EEIO), which is the eastern pole of the IOD and is defined as 10 S-0 S, 90E-110E, with land points and ocean points in Java Sea that lie within this region masked out.

All diagnostics use hindcast data from 1993 onward, except for the time series in Fig. 11 (these start in 1981). For each presented diagnostic, the end of the considered period is set by the data set with the shortest respective reforecast period.

**Table 1:** List and setup of ECMWF hindcast data used in this paper

Name	Initial conditions	Resolution	Model
SEAS5	ORAS5, ERA-Interim/operations	Tco319 L91 ORCA025 Z75	IFS CY43R1, NEMO3.4
SEAS5_LR	ORAS5-LR, ERA-Interim	Tco319 L91 ORCA1 Z42	IFS CY43R1, NEMO3.4
SEAS5_LR_O4	ORAS4, ERA-Interim/operations	Tco319 L91 ORCA1 Z42	IFS CY43R1, NEMO3.4
SEAS5_obsSST	ERA-Interim/operations	Tco319 L91	IFS CY43R1, uncoupled, forced by observed SSTs
SEAS4	ORAS4, ERA-Interim	T255 L91 ORCA1 Z42	IFS CY36R4, NEMO3.0
FC_AtmNudge_Ctrl	ERA5	Tco199 L137 ORCA 1Z75	IFS CY47R3
FC_AtmNudge_5day	ERA5	Tco199 L137 ORCA 1Z75	IFS CY47R3
FC_AtmNudge_2hr	ERA5	Tco199 L137 ORCA1 Z75	IFSCY47R3

## Results

### Description and seasonal evolution of bias in SEAS5 and comparison to SEAS4

**Fig. 2:**
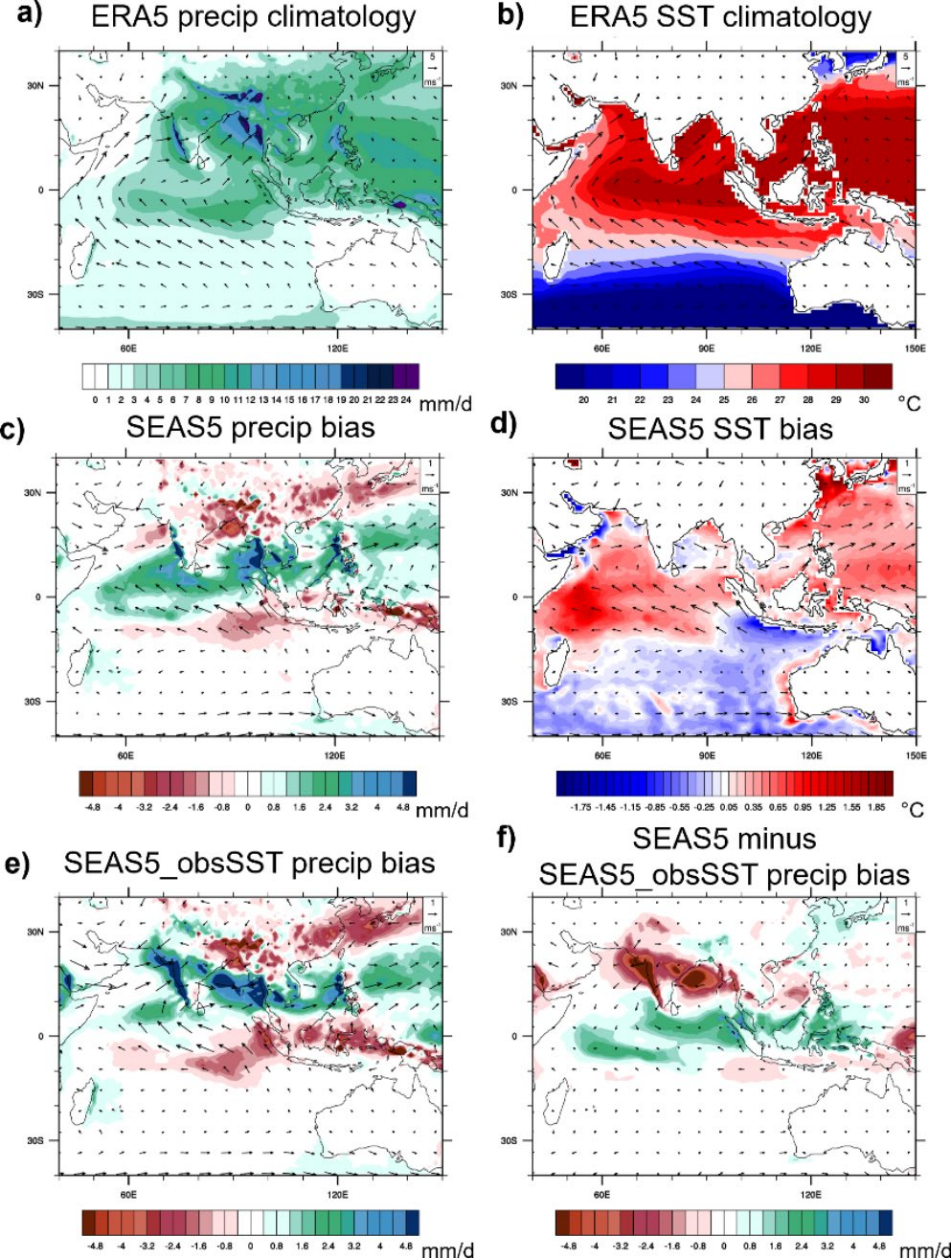
Climatological JJA (**a**) precipitation and (**b**) SST with 10 m winds overplotted as arrows based on ERA5 data. Bias in JJA of (**c**) precipitation and (**d**) SST and associated wind errors of SEAS5 forecasts initialized in May. (**e**) JJA precipitation and wind bias of SEAS5_obsSST forecasts initialized in May; (**f**) Change in JJA precipitation and wind bias from SEAS5_obsSST to SEAS5. All fields are averaged over 1993–2021, and ERA5 is used as a reference for bias estimation

Figure 2a(b) present the JJA seasonal mean precipitation (SST) from ERA5 in the tropical Indian Ocean and Western Pacific together with the mean surface wind circulation. SSTs exhibit the well-known structure with warmest SSTs in the Indo-Pacific warm pool. Strongest precipitation generally occurs where SSTs are highest, and greatly enhanced precipitation can be found close to and over the coastal southeast-Asian continent, where the monsoon circulation leads to strong moisture flux convergence (Mayer et al. 2013). Figure 2c shows that SEAS5 has a negative precipitation bias over the EEIO, and a positive precipitation bias in the north Indian Ocean and Northwest subtropical Pacific. This positive bias is collocated with a positive SST bias in these regions (Fig. 2d; a comparison of SEAS5 and SEAS4 SST bias maps can be found in Johnson et al. 2019). Associated with the precipitation bias, SEAS5 exhibits a too strong anticyclonic circulation over the Northern Indian Ocean. Indian Ocean precipitation in SEAS5 is shifted northward compared to ERA5, yielding a positive (negative) precipitation bias north (south) of the equator. The negative precipitation bias south of the equator is associated with a negative SST bias (Fig. 2d), which is associated with too strong easterlies. The precipitation bias of SEAS5_obsSST (Fig. 2e) exhibits similar patterns as in coupled mode, but the positive bias over the north Indian Ocean is stronger and moved on the adjacent continents compared to the coupled forecasts. The overestimation of the anticyclonic circulation in the Indian Ocean is enhanced in the uncoupled compared to the coupled forecasts, which has implications for the precipitation. Figure 2f compares precipitation from coupled and uncoupled runs directly and confirms that the coupled model produces comparatively more precipitation over the ocean, which is likely related to the developing warm SST bias that acts to reduce the sea/land temperature gradient and hence monsoon circulation.

**Fig. 3:**
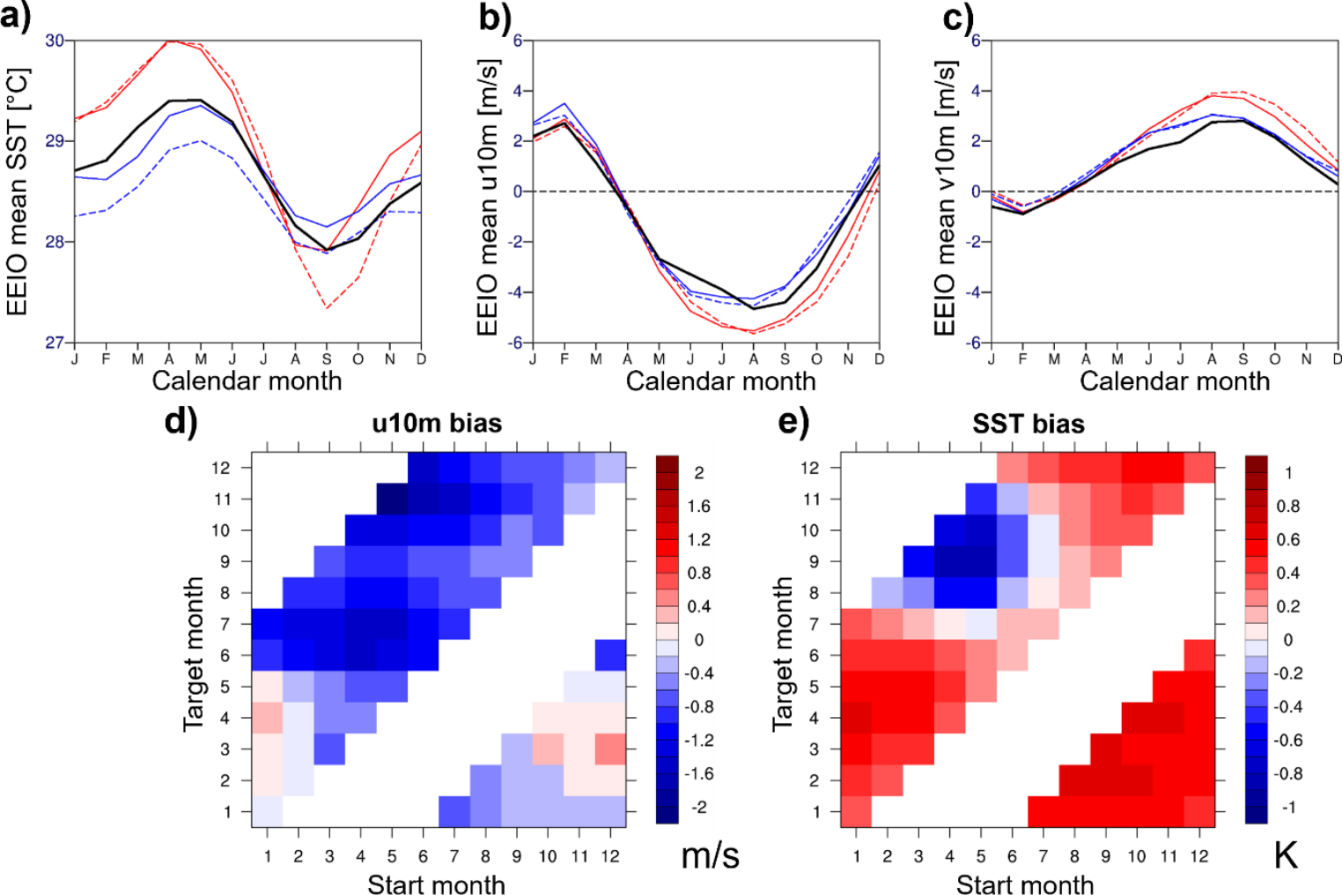
Top row: Monthly climatologies of (**a**) SST, (**b**) 10 m zonal wind, and (**c**) 10 m meridional wind in the EEIO from SEAS5 (red curves) and SEAS4 (blue curves) as a function of target month (full line represent lead months 1–3, dashed lines lead months 4–6); the ERA5-based climatologies are shown in black (as a proxy for observations); The lower row shows the SEAS5 (**d**) 10 m zonal wind and (**e**) SST forecast bias (validated against ERA5) in the EEIO as a function of start months and target month. Plots are based on 1993–2021 data

Figure 3a-c shows observed and forecasted (as a function of target month) annual cycles of SSTs and surface winds in the EEIO. Shown are the forecasts from SEAS5 and SEAS4. Observed SSTs are warmest in April-May (Fig. 3a), coinciding with weak zonal (Fig. 3b) and meridional (Fig. 3c) surface winds. Coolest SSTs are obtained in September, when winds and associated upwelling are much stronger. The wind peaks as part of the Indian and Southeast-Asian monsoon circulation. SEAS5 forecasts exaggerate the warm SST peak in boreal spring and exaggerate the cool peak in boreal fall, especially at 4–6 months lead time (Fig. 3a). The cold SST bias of SEAS5 in fall is associated with an easterly and southerly wind bias (Fig. 3b and c), suggestive of a feedback between easterly winds and oceanic upwelling. SEAS4 has more moderate SST and wind biases in the EEIO, with no cold SST and easterly wind bias in boreal fall.

Figure 3d and e systematically present zonal wind and SST bias in the EEIO from SEAS5 as a function of target month and initialization time. Forecasts started in all calendar months develop an easterly wind bias by June the latest, March-May starts even earlier. SST forecast bias is positive for all target months from January-July, regardless of the initialization time. However, from target month August, forecasts initialized in February-June develop a prominent cold SST bias, as seen already for the May starts in Fig. 2d. Only forecasts initialized from July onward do not develop a cold SST bias. The weak lead-time-dependence of the cold SST bias in the EEIO suggests that the bias is strongly linked with the seasonal cycle, consistent with results from the UK Met Office system by Martin et al. (2021). Only the climatologically cooler SSTs and shallower thermocline in boreal fall allow for a feedback between the easterly wind bias and SSTs in the EEIO (SST_EEIO_) to develop. This feedback is strongly pronounced for May starts. Hence, we will focus on May starts in the rest of this paper.

At this point it is useful to look at the relationship between mean SST bias and mean zonal wind bias in the EEIO in the different C3S models, which is shown in Fig. 1c. A weak easterly wind (positive wind bias) always exists with a warm SST bias. An overly strong easterly wind (negative wind bias) nearly always exists with a cold SST bias. One exception is ECCC-S3, which has a slightly positive wind bias, though some individual months within JJA have a negative wind bias (not shown). While the sign of the SST bias is clearly linked to the sign of the wind bias, there is a lot of variation in the relationship between the magnitude of the zonal wind and SST bias – clearly indicating that other characteristics of the model, beyond the zonal wind bias, determine the size of the SST bias.

### Surface bias evolution in different versions of SEAS5

**Fig. 4:**
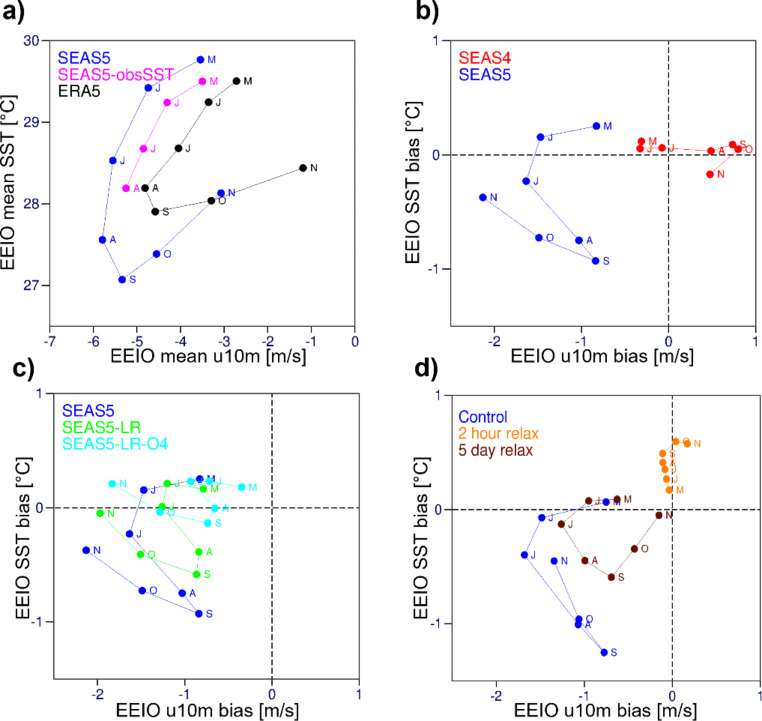
Evolution of 10 m u-wind and SST and its bias in EEIO at different lead times: (**a**) climatologies from SEAS5, SEAS5_obsSST, and ERA5 (1993–2015), (**b**) bias of SEAS5 and SEAS4, (**c**) bias of SEAS5, SEAS5_LR, and SEAS5_LR_O4, (**d**) bias of relaxation experiments: control, 5-day relaxation, 2-hourly relaxation. Bias plots use ERA5 as a reference. All forecast results based on hindcasts initialized in May 1993–2015

Figure 4 compares the temporal co-evolution of zonal wind and SST biases in the EEIO in hindcasts initialized in May. To set the scene, Fig. 4a shows the climatological evolution of the two quantities as represented by ERA5 and SEAS5, and SEAS5_obsSST. It shows that SEAS5 forecasts started in May exhibit an easterly wind bias from lead month 1, while the cold SST bias develops from lead month 3 onward (consistent with Fig. 3d and e). This results in an amplified SST seasonal cycle in SEAS5 w.r.t. ERA5, as well as a shift towards stronger easterlies (consistent with Fig. 3a and b). SEAS5_obsSST also exhibits an easterly wind bias throughout the forecasts, although weaker than in coupled mode, suggesting that the cold SST bias of SEAS5 develops as a result of the easterly bias and then amplifies as a coupled feedback. The cold SST bias of SEAS5 peaks in Aug-Sep-Oct (Fig. 4b). In contrast to SEAS5, SEAS4 (Fig. 4b) exhibits a weaker easterly bias in the first few lead months and develops a westerly bias from August onward. Consistent with this, SEAS4 exhibits a weak negative SST bias only in November. It is also noteworthy that SSTs in the EEIO are biased warm during the first two lead months in SEAS5, which is not the case in SEAS4. This has been attributed to shallower tropical mixed layers and enhanced downwelling longwave radiation in SEAS5 compared to SEAS4 (Johnson et al. 2019). Figure 4c evaluates the dependence of SEAS5 bias evolution to resolution and ocean initial conditions. SEAS5_LR exhibits a qualitatively similar, albeit less pronounced, bias evolution as SEAS5, suggesting that the increased resolution of SEAS5 amplifies the biases. SEAS5_LR_O4 has a strong easterly bias comparable to that of SEAS5, but SST bias remains weakly positive throughout the forecast. This suggests that ORAS4 initial conditions are less susceptible to the wind bias compared to ORAS5 initial conditions.

Figure 4d shows results from the experiments where the atmosphere is nudged towards analyses from ERA5. The control run with no atmospheric nudging (FC_AtmNudge_Ctrl) exhibits qualitatively similar bias evolution as SEAS5. Differences are expected given that FC_AtmNudge_Ctrl differs in many aspects from SEAS5 (lower atmosphere and ocean resolution, more recent IFS cycle including relevant upgrades, e.g., to atmospheric moist physics, and others; see Table 1).

The hindcasts with moderately strong nudging (FC_AtmNudge_5daily) exhibit reduced wind and SST biases compared to the control run up to November. Nevertheless, the very similar wind bias in lead month 1 suggests that atmospheric errors develop rapidly and are likely associated with a fast spinup of precipitation errors in the Northern Indian Ocean and the monsoon areas. Compared to the control, wind and SST biases recover more quickly in Oct-Nov, when monsoon circulation ceases. The strong nudging experiment (FC_AtmNudge_2hrly) keeps the winds close to ERA5 analyses, and SST bias subsequently develops quite differently, yielding a warm bias of ~ 0.6 K by October. This result suggests that the strong constraint of the wind towards ERA5 prevents the excessive shoaling of the thermocline. The presence of a cold thermocline bias in FC_AtmNudge_2hrly cannot be checked as no sub-surface temperatures were archived with this experiment. The warm SST bias in FC_AtmNudge_2hrly is likely related with excessive warming of the mixed layer as a result of the missing feedback with atmospheric fluxes via evaporation, convection, and clouds, which would act to dampen the positive SST tendency.

### Subsurface bias evolution

**Fig. 5:**
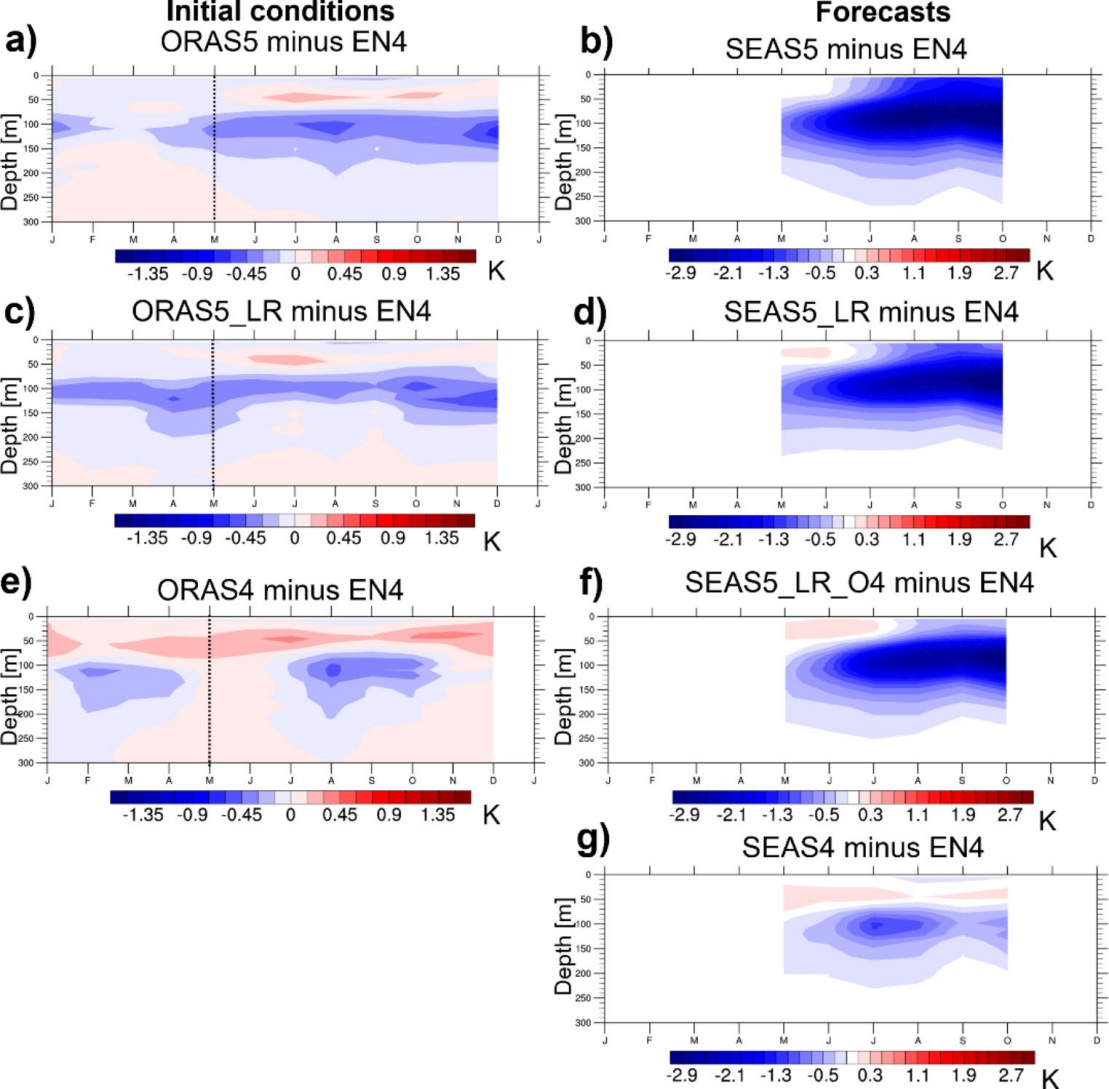
Mean annual cycle of subsurface temperature bias in EEIO of **a**) ORAS5, **c**) ORAS5-LR, and **e**) ORAS4 averaged over 1993–2015. Subsurface ocean temperature bias in EEIO in long-range coupled forecasts from (**b**) SEAS5, (**d**) SEAS5_LR, (**f**) SEAS5_LR_O4, and (**g**) SEAS4 for lead months 1–6 (May starts 1993–2015). Hadley EN4 analyses are used as reference in all figures

Figure 5a shows the mean annual cycle of subsurface temperature bias of ORAS5 in the EEIO, using Hadley EN4 (Good et al. 2013) data as a reference. ORAS5 exhibits a cool bias order ~ 0.5 K around the thermocline almost year-round, with a maximum around 100 m depth. Above the thermocline, there is a weak warm bias from June to December; below 150 m the biases are small. Figure 5b shows the lead-time dependent subsurface temperature bias in SEAS5 initialized in May 1993–2015. In month-1, SEAS5 has a moderate cold bias of ~-0.5 K peaking at ~ 100 m depth, which is largely consistent with the temperature biases found for ORAS5 (Fig. 5a). The negative bias at that depth rapidly amplifies with lead time and becomes lower than − 3 K from July onward. From July onward, the bias also extends to the surface, consistent with Figs. 3e and 4b. The forecast bias below ~ 200 m remains small at all lead times.

To explore the impact of ocean model resolution and versions as well as the cycle of the atmosphere model, the other panels of Fig. 5 show analogous diagnostics for different ocean reanalyses and forecasts. ORAS5_LR, a low-resolution version of ORAS5 ran at ORCA1_Z42, shows a very similar annual cycle of subsurface temperature bias (Fig. 5c) as ORAS5. Figure 5d shows the subsurface temperature bias of a low-resolution version of SEAS5 (SEAS5_LR, see Table 1) that is initialized from ORAS5_LR. Consistent with the similar sub-surface biases of ORAS5 and ORAS5_LR, SEAS5-LR exhibits a similar bias as SEAS5 in lead month 1. The negative bias around the thermocline subsequently amplifies, but not as pronounced as in SEAS5. Also, the negative bias close to the surface is substantially reduced compared to SEAS5. The reduced surface impact of the cold thermocline bias in SEAS5_LR is consistent with the reduced SST bias and, as a result, a reduced u10m bias compared to SEAS5 (compare Fig. 4c).

ORAS4 has a reduced EEIO cool bias at 100 m compared to ORAS5_LR, which only appears in a few calendar months (February and March, August and September). In return, ORAS4 exhibits a warm bias in the mixed layer (Fig. 5e). Figure 5f shows the subsurface temperature bias of a low-resolution version of SEAS5 that is initialized from ORAS4, with the same coupled model cycle used in SEAS5 and SEAS5_LR (SEAS5_LR_O4, see Table 1). The negative bias with a maximum around the thermocline is also present for this experiment, but with a magnitude that is further reduced compared to SEAS5_LR. This is most visible in lead month 1, which is consistent with the warmer thermocline temperatures of ORAS4 compared to ORAS5_LR (compare Fig. 5c and e). More importantly, in SEAS5_LR_O4 the cold bias appears later at the surface and is much reduced compared to SEAS5 and also to SEAS5_LR. An important consequence is that the subsurface errors in SEAS5_LR_O4 are fairly uncoupled from the atmosphere in boreal summer, during the monsoon season. Figure 4c confirms the positive impact of ORAS4 initial conditions on surface biases, with only small negative SST bias in September-October, despite the strengthening easterly wind bias.

Finally, Fig. 5g shows the subsurface temperature bias of SEAS4 forecasts initialized in May. It develops a moderate cold thermocline bias (peaking in July with a subsequent decline), which is much reduced compared to SEAS5 and does not affect the mixed layer and SST. This also represents a remarkable difference compared to SEAS5_LR_O4, given the two forecast data sets use the same ocean initial conditions and only differ in the ocean model version (NEMO3.0 versus NEMO3.4, see Table 1) and the atmospheric model cycle used in the forecasts. The effect of the latter is deemed minor, given that an experimental version of SEAS4 using observed SSTs exhibits a similar, albeit slightly reduced, easterly wind bias as SEAS5_obsSST (-0.63 vs. -0.75 m/s for May starts in JJA 1993–2015).

We conclude that all considered forecast experiments that use NEMO3.4 show a strong cold bias developing around the thermocline for May starts, and in all the cold bias reaches the surface by boreal summer and thus induces a cold SST bias as well, albeit at varying degrees. The biases are aggravated by the higher horizontal and vertical resolution (note the Z42 and Z75 configurations have 10 m and 1 m vertical resolution in the top layers, respectively) in the upper in the ocean.

The difference in forecast biases between SEAS5-LR and SEAS5-LR-O4 is due only to the ocean initial conditions, since they use the same model setup in forecast mode. One important difference between ORAS5 and ORAS4 are the different ocean model versions used for the long reanalyses (NEMO3.4 and NEMO3.0, respectively), which suggests that the ocean model version can impact the forecasts alone through the initial conditions. However, we emphasize that ORAS5 and ORAS4 differ in several other aspects, which potentially additionally contribute to the differences between SEAS5-LR and SEAS5-LR-O4.

**Fig. 6:**
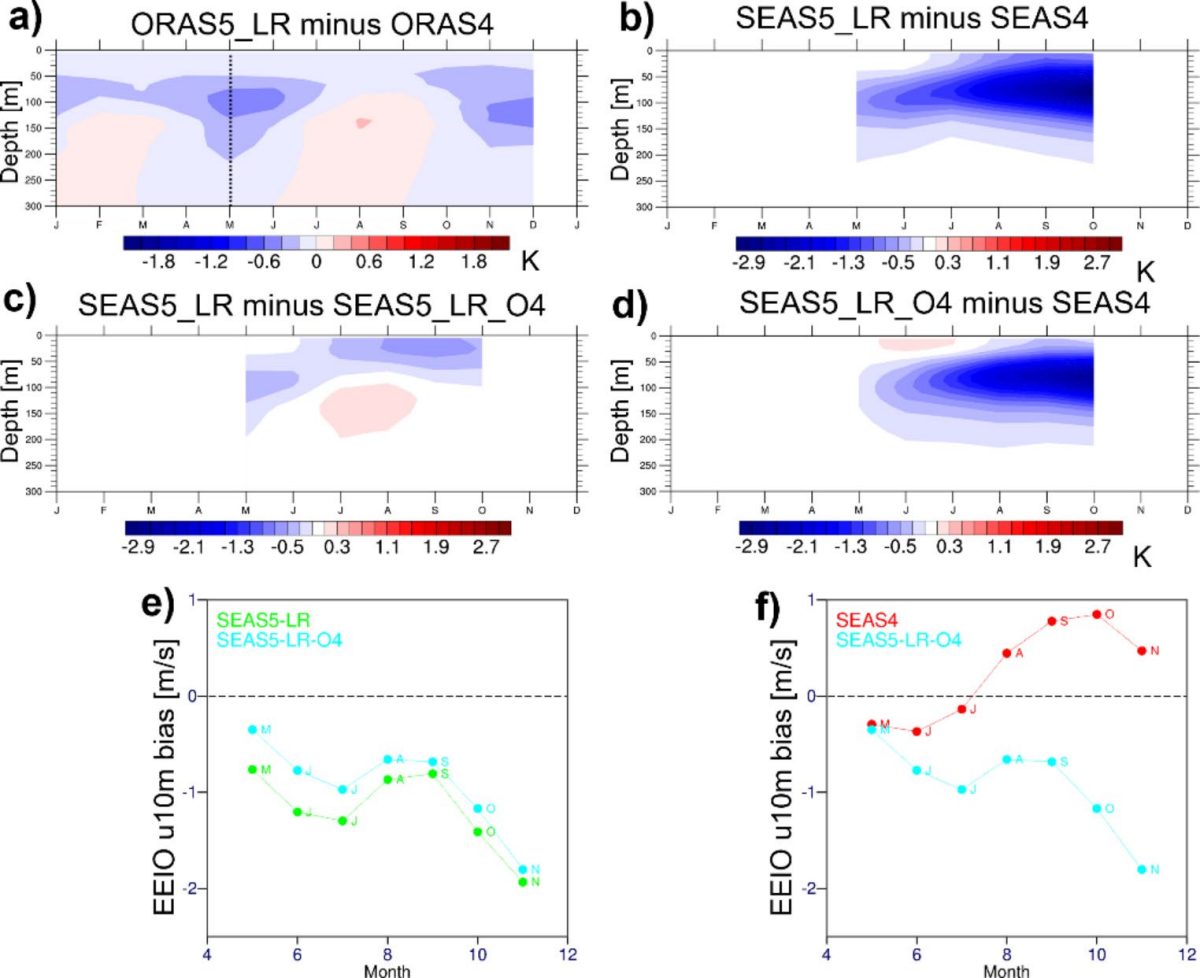
**a**) Difference in mean annual cycle of subsurface temperature bias in EEIO between ORAS5-LR and ORAS4; Difference in subsurface ocean temperature bias in EEIO between **b**) SEAS5_LR and SEAS4, (**c**) SEAS5_LR and SEAS5_LR_O4, and (**d**) SEAS5_LR_O4 and SEAS4 for lead months 1–6 (May starts 1993–2015); (**e**) and (**f**) show the evolution of EEIO wind bias in the experiments shown in (**c**) and (**d**), respectively

Figure 6 further presents an (approximate) decomposition of the total effect of (i) the ocean initial conditions and (ii) the ocean/atmospheric model version on the subsurface forecast bias. Figure 6a shows the differences between ORAS5-LR and ORAS4 initial conditions. Figure 6b shows the total effect represented by SEAS5_LR minus SEAS4. The contributions from the ocean ICs (SEAS5_LR minus SEAS5_LR_O4) is shown in Fig. 6c; and the impact of the forecast model version is shown in Fig. 6d (SEAS5_LR_O4 minus SEAS4). Comparison between Fig. 6c and d reveals that the substantially larger effect in the thermocline stems from the model version during the forecast, while the difference in initial conditions (ORAS5-LR and ORAS4 were created with different model versions) in the thermocline is hardly amplified when using the same forecast model. However, the change from ORAS4 to ORAS5-LR initial conditions yields a relatively strong surface cooling of the forecasts. This is consistent with ORAS5-LR being cooler than ORAS4 above the thermocline (Fig. 6a), and it is likely a consequence of the different vertical mixing formulations in the respective ocean model versions used to create the reanalyses. Figure 6e and f additionally show the wind biases in the two experiments shown in Fig. 6c and d, respectively. Figure 6e illustrates the fast time scales of the feedback between the atmosphere and the mixed layer, with the different ocean initial conditions creating differences in the wind already at lead month 1. Figure 6f demonstrates clearly the feedback operating at longer time scales typical of the thermocline: the easterly wind bias in SEAS5_LR_O4, similar to that of SEAS4 in the first month, gets amplified once the subsurface bias reaches the surface.

### State-dependence of subsurface errors

**Fig. 7:**
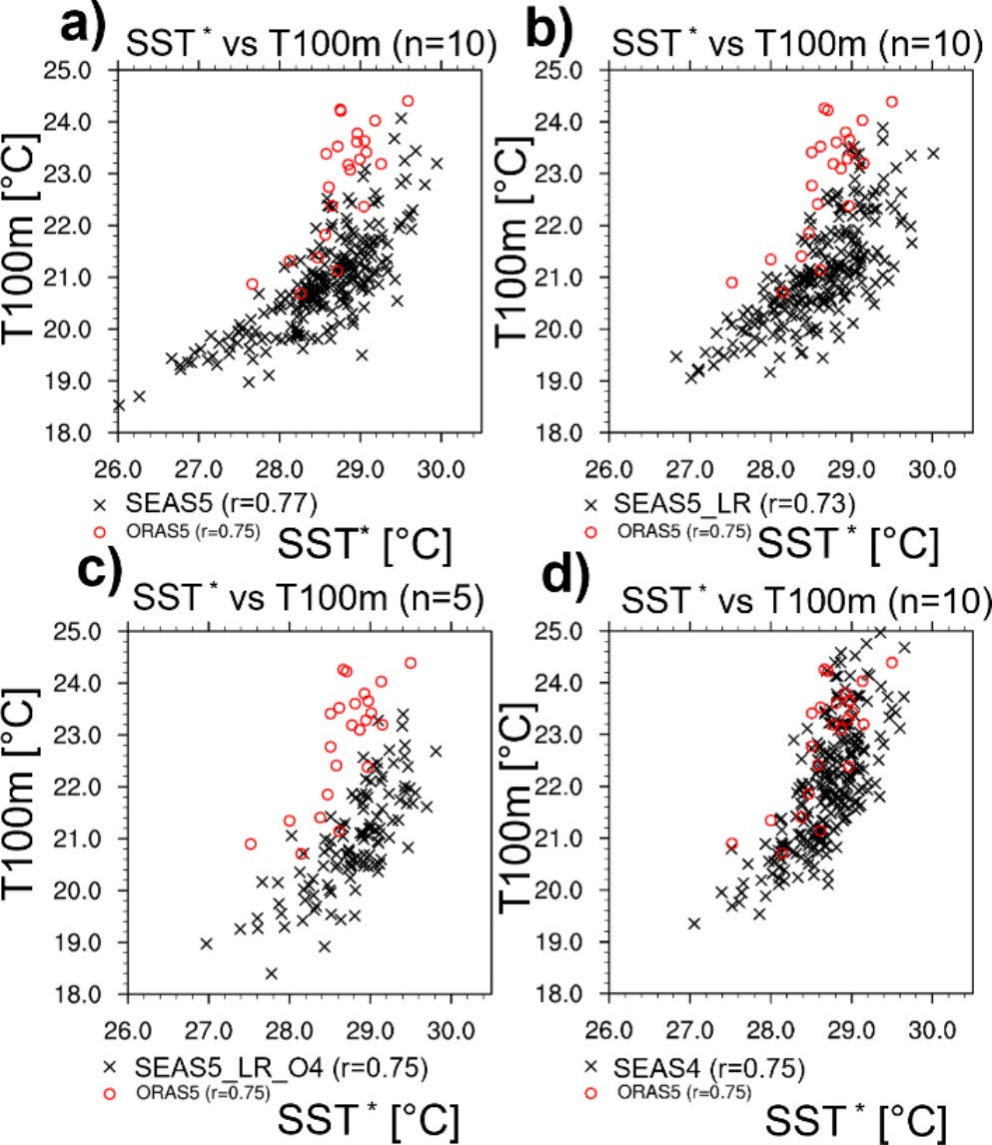
Scatter diagrams of JJA SSTs^*^ in EEIO against temperature in 100 m depth in JJA in EEIO. Black crosses show single members of (**a**) SEAS5 (1993–2015) and (**b**) SEAS5-LR (1993–2015), (**c**) SEAS5-LR-O4 (1993–2015), and SEAS4 (1993–2015) forecasts (May starts). Red circles show relationships based on ORAS5. SST^*^ represents temperature of the uppermost vertical level of the respective data set to allow for consistency with T100

While the previous section focused on the subsurface bias, we now turn to the state-dependent relationships between SSTs and sub-surface temperatures in the EEIO. Although biases are maximal in August-October (ASO), we show results for JJA to focus on their development. We also note that the revealed relationships for JJA are in qualitative agreement when considering only single months June, July, or August. Figure 7 shows the joint distribution of SST^*^ and temperatures in 100 m depth (T100) in EEIO in JJA (the data are presented as heat maps in S1) for the different initial conditions and forecast experiments. For consistency with subsurface temperatures, SST^*^ represents ocean temperatures of the uppermost level rather than the surface temperature used otherwise in this paper. T100 can be interpreted as a proxy for thermocline depth, with high values indicating a deep thermocline and vice versa.

As expected, the scatter plots show a positive correlation between SSTs^*^ and T100 in EEIO. The observed JJA distribution suggests the existence of two regimes. The slope is steeper in the warm regime (for SST^*^>Tc, a threshold temperature of ~ 28.5 °C) with strong T100 variations (~ 21.5 to 24.5 °C in ORAS5, red circles in Fig. 7a) compared to a relatively small range in SST^*^ (~ 28.5 to 29.5 °C), indicative of a weak thermocline feedback in the warm regime, when the thermocline is deep. The steep slope in the warm regime can also be interpreted as little SST^*^ sensitivity to variations of a relatively deep thermocline, together with the fact that deep atmospheric convection prevents the growth of SST^*^ perturbations (see also discussion in Sect. 3.5.2). We note that in ORAS5 the cold regime is a rare event and represented only by very few cases, but its existence is supported by the distribution based on forecasts (discussed below), which enter the cold regime more frequently.

In the cold SST^*^ regime (SST^*^ < Tc), the slope is relatively flat with a smaller change in subsurface temperatures being associated with a larger change in SST^*^. This is indicative of a shallower thermocline (maximum vertical gradient in shallower layers than 100 m), and a strong thermocline feedback (comparatively small variations of subsurface temperature can induce large variations of SST^*^). The flatter slope of the cold regime can be interpreted as a strong sensitivity of the SST^*^ to variations of a shallow thermocline, a sensitivity that is not capped by atmospheric feedbacks due to the weaker local air-sea interaction in the cold regime. For example, due to the absence of deep convection in the cold regime (see also Sect. 3.5.2), there is a weaker cloud and hence surface radiation feedback that could damp SST^*^ perturbations.

The T100/SST^*^ distribution based on SEAS5 forecasts is shifted vertically compared to ORAS5 (Fig. 7a), i.e., T100 for a given SST^*^ is lower in SEAS5 compared to ORAS5. Nevertheless, SEAS5 exhibits a similar distinction of regimes around SST^*^=Tc. However, in contrast to ORAS5, a much larger fraction of forecasts resides in the cold regime (see also S1b), and very cool T100 values are obtained by some members which have never been observed during the considered period. This is consistent with a shallowing error of the thermocline in the forecasts.

Figure 7b shows the T100/SST^*^ relationship for SEAS5_LR compared to ORAS5. SEAS5_LR has a similar cool T100 bias as SEAS5 but has a weaker tendency to go into the cold regime. The long tail for cold SST^*^ is reduced in SEAS5_LR, and the relative frequency of high SST^*^ (> 29 °C) is higher compared to SEAS5 (see also S1c).

A further gradual change in behavior can be seen for SEAS5_LR_O4 (Fig. 7c). This experiment exhibits a less pronounced distinction between warm and cold regime compared to SEAS5, i.e., the flattening of the T100/SST^*^ relationship for SST^*^<Tc is less present, and cool T100 values below 19 °C are reached for comparatively warm SSTs^*^. Conversely, in SEAS5_LR_O4, low T100 values do not translate into SSTs^*^ as low as in SEAS5. This is consistent with the result that the unconditional cold subsurface bias in SEAS5_LR_O4 does not appear at the surface as prominently as in SEAS5 (compare Fig. 5f). Another salient difference of SEAS5_LR_O4 with SEAS5 are the increased frequencies of cases in the warm regime (not shown explicitly), but it has to be kept in mind that SEAS5_LR_O4 consists of only 5 ensemble members, which makes robust statements about changes in forecast distributions difficult.

Finally, the joint forecast distribution of T100 and SST^*^ from SEAS4 is closest to ORAS5 (Fig. 7d), with a reduced negative offset of T100 and a non-existent long cold tail of SSTs^*^ and T100. SST^*^/T100 relationships for ASO generally agree with the above findings and tend to show the described features, including the two distinct regimes, in an even more pronounced manner (not shown).

### Atmospheric feedback and remote impact

#### Atmospheric response to SSTs in the EEIO

**Fig. 8:**
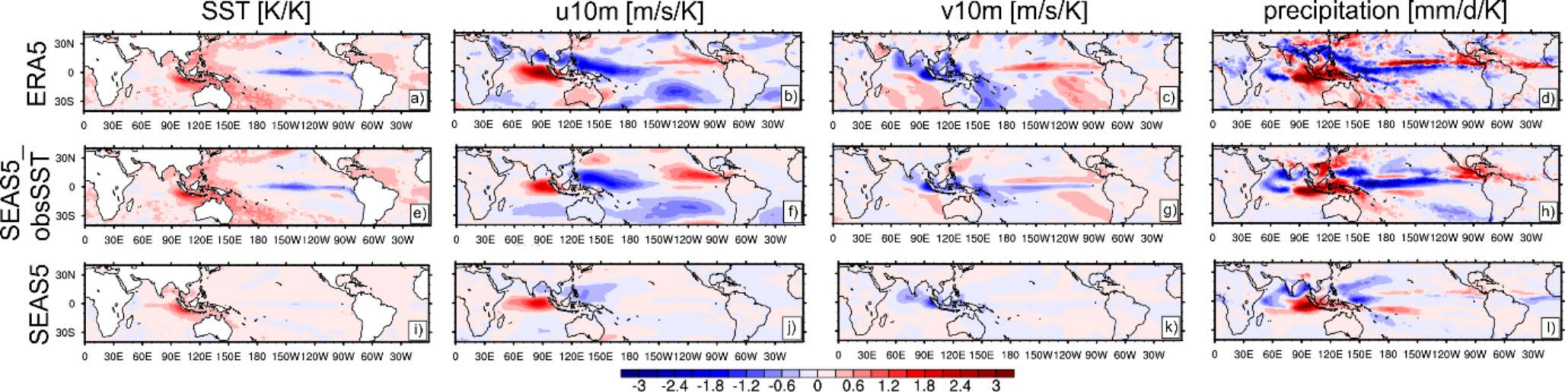
Regression of JJA (first column) SST fields, (second column) 10 m u-wind fields, (third column) 10 m v-wind fields, (fourth column) precipitation fields onto SST in JJA averaged over the EEIO region, based on (first row) ERA5, (second row) SEAS5_obsSST, and (third row) SEAS5 (forecasts initialized in May 1993–2021)

Figure 8 presents relationships between fields of SSTs, 10 m zonal wind, 10 m meridional wind, and precipitation with SST_EEIO_, represented by contemporaneous linear regression coefficients of forecasts for JJA. The first row of Fig. 8 shows relationships as diagnosed from ERA5. For a given warm anomaly in the EEIO, there is a weak cold anomaly in the Western Equatorial Indian Ocean (WEIO), reflecting the anti-correlation arising from the IOD mode. Furthermore, a positive SST_EEIO_ anomaly tends to be associated with a La Niña – like state in the tropical Pacific. Along the equatorial Indian Ocean, there is a westerly wind anomaly associated with a warm SST_EEIO_ anomaly, while there is an easterly wind anomaly in western Pacific reaching into the northern subtropical Indian Ocean. Furthermore, there is a northerly wind anomaly in the northern subtropical Indian ocean in association with a warm SST_EEIO_ anomaly, reflective of a weakened flow into the monsoon regions. This is also confirmed by the regression of precipitation with SST_EEIO_, which shows reduced precipitation over India and southeast Asia. Furthermore, precipitation in the EEIO is enhanced and reduced in the WEIO with a positive SST_EEIO_ anomaly. The precipitation regression pattern over the Pacific is typical for a La Niña – like state (Clarke 2008; Mayer et al. 2013).

Regression fields based on SEAS5_obsSST (second row of Fig. 8) show several differences with those based on ERA5. Most notably, the negative wind regression coefficients (indicative of westerly winds) along the equatorial Indian Ocean is weaker with reduced spatial extent. The easterly wind signal in the Western Pacific is reproduced well, but the extension into southeast Asia is missing. Consistent with this, the observed precipitation signal over southeast Asia and India is not reproduced by SEAS5_obsSST, and the northerly wind signal in the northern Indian Ocean is much reduced compared to ERA5.

The last row of Fig. 8 shows the regression patterns for SEAS5. The SST relationship with SST_EEIO_ around the Maritime continent is in good agreement with ERA5, but the zonal structure of regression coefficients along the equatorial Indian Ocean is not accurately represented (positive coefficients extending too far to the west). In the Pacific, the SST relationship with SST_EEIO_ is generally weak: the warm SST anomalies in the Warm Pool and the cold SST in the eastern equatorial Pacific seen in ERA5 are hardly visible in SEAS5, indicating a weaker link between IOD and ENSO in the coupled model compared to observations. Consistently, the wind regression pattern in the Pacific differs from that in ERA5 and SEAS5_obsSST. Nevertheless, the negative regression coefficients (indicative of easterly wind anomalies) in the northwestern subtropical Pacific extend much further to the west than in SEAS5_obsSST, which indicates a better performance of the coupled forecasts in that regard. Also, the precipitation response in the monsoon regions is improved compared to SEAS5_obsSST, although still too weak compared to ERA5. Along the equatorial Indian Ocean, the west-east dipole in the precipitation regression onto SST_EEIO_ is less pronounced than in the reanalysis. We stress that not all differences between the regressions using SEAS5 and those using ERA5 are attributable to a biased response of the atmosphere to SST_EEIO_, since there are strong two-way interactions across tropical basins (Cai et al. 2019), which includes errors. For example, the missing signature of La Niña in Fig. 8i suggests a lack of ENSO-forcing of the circulation in the Indian Ocean via changes in the Walker Circulation.

#### EEIO atmospheric response to local SSTs

**Fig. 9:**
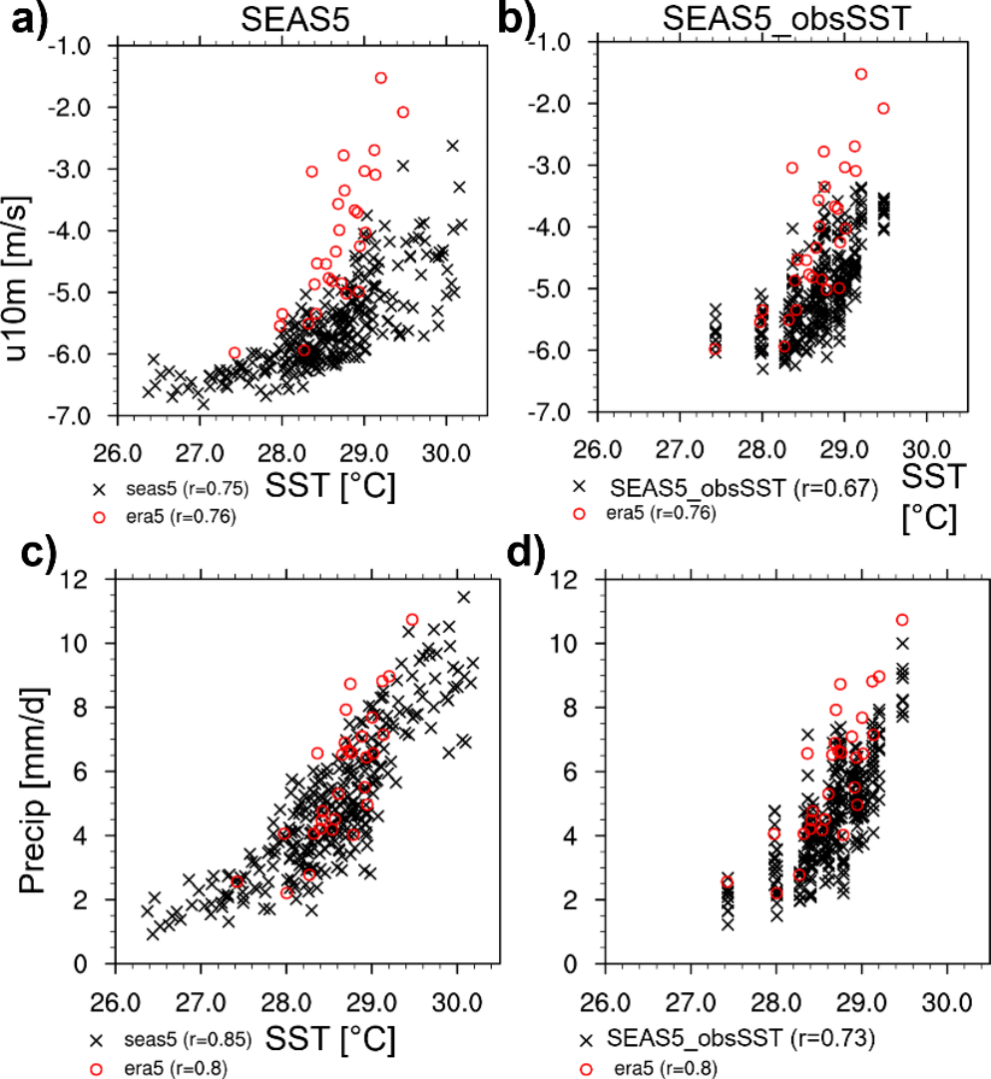
Scatter diagrams of JJA SSTs in EEIO against (**a**,**b**) 10 m u-wind and (**c**,**d**) precipitation in JJA in EEIO. Black crosses show single members (10 per start date) of (**a**,**c**) SEAS5 and (**b**,**d**) SEAS5-obsSST forecasts initialized on 1 May 1993–2021 and red circles relationships based on ERA5 1993–2021

Figure 9 shows scatter diagrams of SST_EEIO_ versus (first row) zonal wind and (second row) precipitation in the EEIO for forecasts and ERA5 in JJA (the zonal wind data are presented as heat maps in S2). The observed SST/wind relationship (Fig. 9a) exhibits a non-linear behavior, with two different regimes for SST above and below Tc = ~ 28.5 °C: the sensitivity of wind to SST variations is higher (steeper slope) in the warm regime.

Since the winds and SSTs are coupled two-ways, the flattening of the u10m/SST relationship in the cold regime can also be interpreted as an increased SST sensitivity to wind variations. In the observations (Fig. 9a), most of the points are in the warm regime, showing a strong reduction of the prevailing easterlies in association with warmer SSTs. SEAS5 forecasts (Fig. 9a) exhibit three major differences w.r.t. reanalyses. First, for a given SST, the easterlies are too strong. Second, the easterly wind bias increases with SSTs (warm regime slope error). Third, SEAS5 SST_EEIO_ is more populated in the cold regime, with a sizable fraction of forecasts reaching much cooler values than observed (see also Fig. S2b). The SST/wind relationship in SEAS5_obsSST (Fig. 9b) is somewhat improved compared to SEAS5, with forecasts lying closer to ERA5 for SSTs < Tc. However, at higher SSTs the warm regime slope error appears similar in coupled and uncoupled forecasts.

The modelled SST/precipitation relationships are closer to observations (compared to the u10m/SST relationship) for both coupled (Fig. 9c) and uncoupled forecasts (Fig. 9d). They show increased precipitation sensitivity for SSTs > Tc, when deep convection typically starts. The relationship in Fig. 9c and d could also be interpreted as deep convection damping further growth of very warm SSTs. Both forecasting systems exhibit a slight underestimation of precipitation at high SSTs, where SEAS5 exhibits weaker underestimation of precipitation at high SSTs (-0.8 mm/d for all cases with SST > Tc and − 1.1 mm/d for all cases with SST > Tc and lower the observed maximum SST) than SEAS5_obsSST (-1.5 mm/d for all cases with SST > Tc). For the cool SSTs only present in SEAS5, the SST/precipitation relationship weakens as precipitation goes towards zero.

#### EEIO atmospheric response to zonal SST gradients

**Fig. 10:**
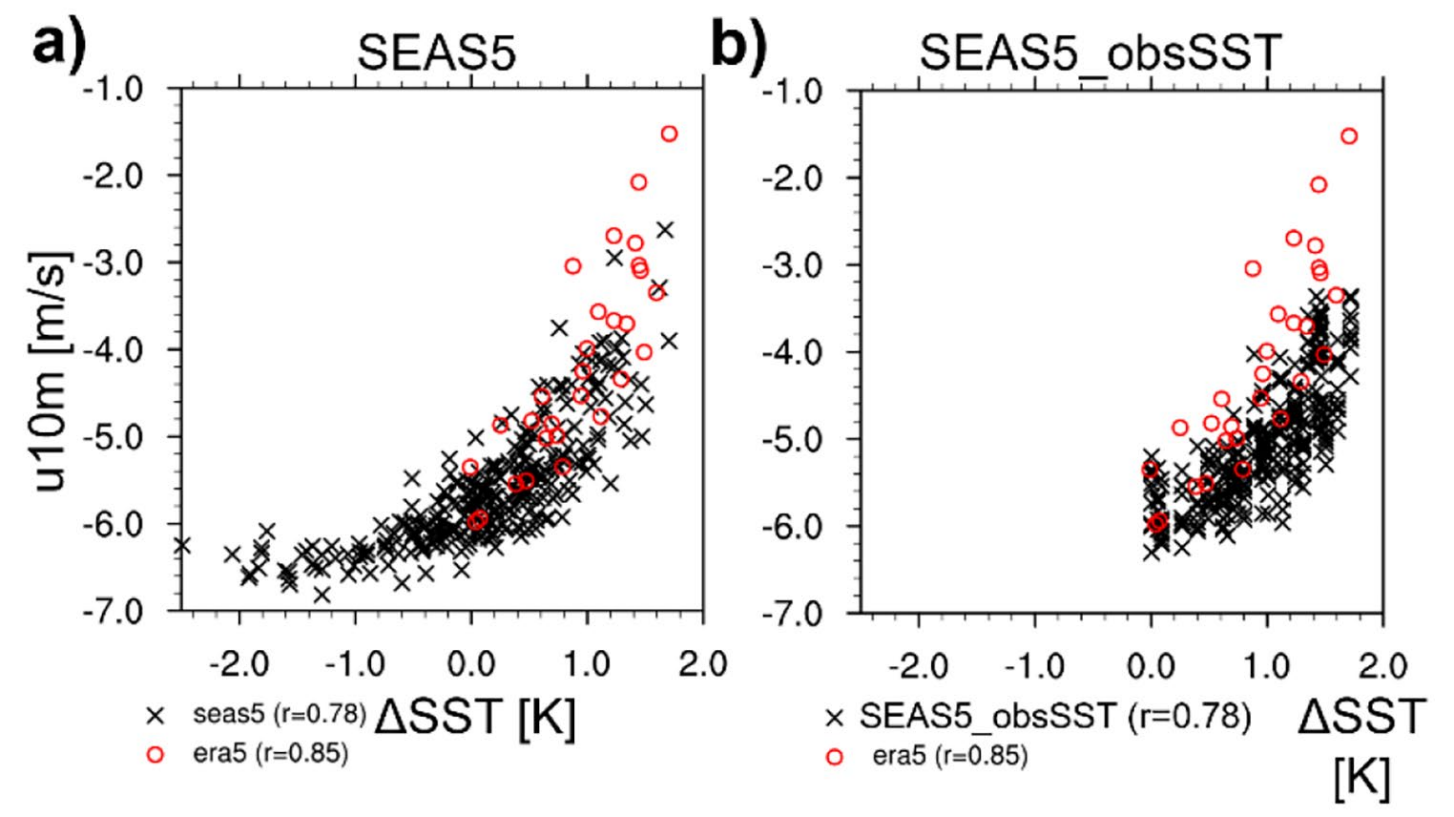
Scatter diagrams of JJA west-east SST gradients (defined as difference SST_EEIO_-SST_WEIO_) in the Indian Ocean against 10 m u-wind in JJA in EEIO. Black crosses show single members (10 per start date) of (**a**) SEAS5 and (**b**) SEAS5_obsSST forecasts on 1 May 1993–2021 and red circles relationships based on ERA5 1993–2021

Next, we look at relationships of u10m and precipitation in the EEIO with the zonal SST gradient across the Indian Ocean, approximated by the difference SST_EEIO_-SST_WEIO_ (ΔSST, where WEIO is defined as 10 S-10 N, 50-70 E). Note that ΔSST differs from the Indian Ocean Dipole Model Index as the latter uses standardized SST anomalies and has the sign reversed (Saji et al. 1999). Figure 10 is analogous to Fig. 9, but now with ΔSST on the x-axis (the data are presented as heat maps in S3). It stands out that the observed zonal SST gradient is always positive and ranges between 0 and 2 K, i.e., SSTs in the EEIO are always warmer than those in the WEIO. In SEAS5 (Fig. 10a), the SST gradient can attain large negative values lower than − 2 K. For the cases where SEAS5 has a positive zonal SST gradient, the ΔSST/u10m relationship in SEAS5 agrees reasonably well with ERA5, although the weakening of the winds in association with a positive SST gradient still seems underestimated in terms of slope. Hence, it appears as if the model captures comparatively well the relationship between zonal winds and zonal SST gradients in spite of the deficient relationship with local SSTs in the EEIO. This can be understood by noting the positive SST bias of SEAS5 in the WEIO in JJA (compare Fig. 4a in Johnson et al. 2019): For a given positive SST_EEIO_ anomaly in SEAS5, ΔSST is smaller compared to observations because of the warm SST_WEIO_ bias (compare also Fig. A2 in Mayer et al. 2022). Indeed, ensemble members with comparatively cool (warm) SSTs in the WEIO exhibit an improved (degraded) wind/SST relationship in the EEIO (see S4). We conclude that the warm regime slope error of SEAS5 (underestimation of weakening of zonal wind in association with warming of SST_EEIO_; Fig. 9a) at least partly results from the warm SST_WEIO_ bias and the fact that the zonal winds are more strongly related to ΔSST rather than SST_EEIO_. However, from Fig. 10a (and S3b) it is also evident that cases with more than moderately positive ΔSST are relatively rare in SEAS5, as the majority of SEAS5 forecasts cluster around neutral SST gradients (even more so in ASO, not shown), which is rarely observed (not to mention the occurrences of negative ΔSST).

SEAS5_obsSST (Fig. 10b) exhibits a similar ΔSST/u10m relationship as SEAS5, with reduced wind spread at SST gradients > 1 K. In the reanalyses, the ΔSST/u10m relationship has a stronger linear slope for positive SST gradients, i.e., the model winds in SEAS5_obsSST do not weaken sufficiently with stronger positive SST gradients (despite the by construction unbiased SST_WEIO_ - in contrast to SEAS5).

The too weak wind response in SEAS5_obsSST for ΔSST > 1 K (Fig. 10b) may be related to the too weak precipitation response for high SSTs in the EEIO (Fig. 9d), which conversely provides too weak diabatic heating and low-level wind convergence. Another aspect is the positive precipitation bias of SEAS5_obsSST over southeast Asia [Fig. 2e; see also Stockdale et al. 2018], which is associated with strengthened equatorial easterlies through the enhanced anticyclonic atmospheric circulation over the northern Indian Ocean. Furthermore, the observed anti-correlation between SST_EEIO_ and southeast-Asian precipitation (i.e., higher SST_EEIO_ goes with reduced precipitation over southeast Asia and vice versa in reanalysis) is not reproduced by SEAS5_obsSST (compare Fig. 8d and h), i.e., the positive southeast-Asian precipitation bias (as shown in Fig. 2e) and associated circulation response is especially strong for high SST_EEIO_, which likely contributes to the conditional easterly wind bias of SEAS5_obsSST in the EEIO. In addition to this, the too weak slope of the ΔSST/wind relationship of SEAS5_obsSST may also indicate a persistent and stable easterly regime which is difficult to destabilize by either local convection or large-scale SST gradients.

We reiterate the coupled nature of the problem, where an easterly wind error initiates a negative SST gradient error and vice versa. However, the additional experiments with prescribed SSTs and nudged atmosphere help to disentangle the picture. The results using SEAS5_obsSST show similar features as the coupled runs, which clearly indicates a biased response of the atmospheric model to changes in the zonal SST gradient, although it should be noted that other larger scale drivers like monsoon precipitation play an additional important role in driving equatorial easterlies. On the other hand, the wind/SST relationships in the forecasts with nudged atmosphere are in very good agreement with observations (see S5). These results suggests that a root cause of the problem lies in the atmosphere, but oceanic feedbacks (which seem overly strong in SEAS5, see previous sections) amplify errors.

### Non-stationarity of EEIO bias

**Fig. 11:**
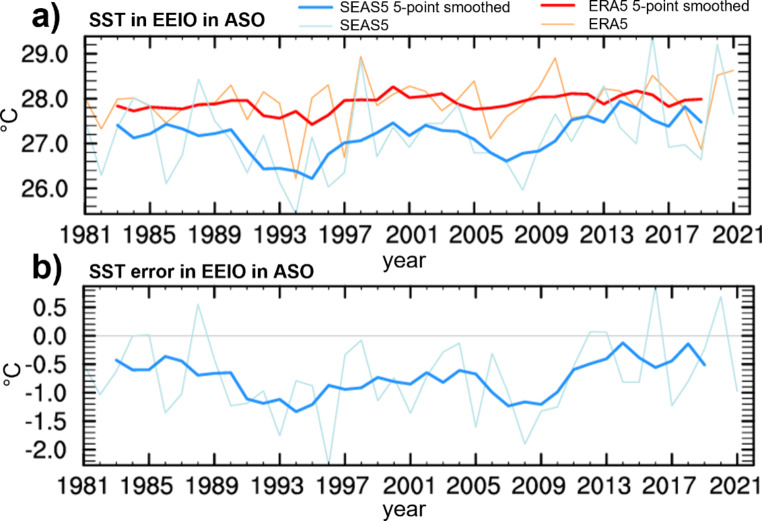
**a**) Time series of ASO SST analyses from ERA5 and forecasts from SEAS5 (ensemble means) and (**b**) their error averaged over the EEIO (May start dates). Lightly colored lines represent annual values, while darker lines represent 5-yearly smoothed series

Previous sections focused on forecast errors averaged across the hindcast period 1993–2021. To add another facet to the problem, Fig. 11 assesses temporal variations of the EEIO SST forecast bias. To infer robust statistical relationships, we here use 25 ensemble members from May start dates 1981–2021. Figure 11a shows time series of observed and forecasted SST_EEIO_ averaged over ASO, the peak season of the Indian Ocean Dipole. Observations exhibit strong interannual variability of SST_EEIO_ (standard deviation SD = 0.54 K) associated with IOD events, but the 5-yearly smoothed observational series suggests that longer-term variations in SST_EEIO_ are moderate (SD = 0.18 K). Ensemble mean SST forecasts from SEAS5 (May starts) show increased interannual variability (SD = 0.85 K) compared to observations. This behavior is consistent with Cai and Cowan (2013) and McKenna et al. (2020) who found stronger IOD activity in models with cold SST bias in the EEIO. The difference between observed and forecast variability is even more pronounced for multi-year variations, with a more than doubled variability of the 5-yearly smoothed forecast time series (SD = 0.39 K) compared to observations (SD = 0.18 K). Forecasted SSTs in the 1990 and 2000s were generally lower than in the most recent decade and, albeit less pronounced, in the 1980s. The low-frequency variations of forecasted SST_EEIO_ result in variations of the errors (see Fig. 11b), with a significantly stronger negative bias during the middle period (-0.97 K during 1993–2011) compared to the recent period (-0.34 K during 2012–2021; p-value < 1e-13). From around 2012 onward, forecast SSTs warm on average and get closer to observations. Inspection of upper ocean heat content in the EEIO from ORAS5 reveals that May initial conditions exhibit qualitatively similar low-frequency variability as the SST forecast error (not shown). How much of the variations in ORAS5 represent true variability or arises from changes in the observing system is unclear. In any case, the non-stationarity of the SEAS5 forecast bias demonstrates that changing background conditions such as multiannual changes in upper ocean heat content in the Indian ocean, but potentially also in remote regions, affect temporal stability of the forecast errors. Moreover, the correlation between ensemble mean forecasts of SST_EEIO_ and their error is highly significant (r = 0.77, p < 10^− 8^), i.e. small real multi-annual variations in the state of the IOD are vastly exaggerated by the hindcasts and indicate state-dependence of SEAS5 forecast errors in the EEIO.

## Summary and conclusions

This paper explores the lead-time-dependent evolution of atmospheric and oceanic forecast errors of SEAS5 in the Indian Ocean, most notably the cold SST and easterly wind bias in the EEIO. This represents one of the few areas of degradation of the forecast system compared to its predecessor SEAS4. The presence of similar problems in several other seasonal forecast systems provided through C3S and the important role of the EEIO for teleconnections urge for a better understanding of the underlying problems.

Analysis reveals that errors are present already in the ocean initial conditions from ORAS5 to some extent, with a too cold thermocline in the EEIO. The subsurface temperature bias in ORAS5 is increased compared to ORAS4, which provides initial conditions for SEAS4. Oceanic resolution has been found to play a relatively small role in this context, which suggests that the different versions of the NEMO ocean model (NEMO3.4 compared to 3.0) along with differences in assimilated data and assimilation methods leads to the increased bias in ORAS5.

Exploration of different forecast experiments allowed isolation of the role of ocean initial conditions, the coupled forecast model, and oceanic resolution in the increased SST forecast bias in SEAS5 compared to SEAS4. We found that a low-resolution configuration of SEAS5 initialized from ORAS4 (SEAS5_LR_O4) already produces a large fraction of the bias increase found in SEAS5 (compared to SEAS4), emphasizing the role of the coupled model version. Use of ORAS5 initial conditions and the higher operational resolution of SEAS5 forecasts further increase the bias. While the strong thermocline bias seems to be associated with the coupled model version, the ocean initial conditions seem to influence the surface expression of the error, with forecasts initialized from the ORAS5-family producing colder temperatures in the upper 50 m than the ORAS4 based forecasts.

Results from uncoupled atmospheric seasonal forecasts (SEAS5_obsSST) and results from Mayer et al. (2022) indicate that within the first few forecast days the atmospheric model establishes an easterly wind bias in the EEIO (and other tropical regions) in the absence of sufficient observational constraints, which at longer lead times further strengthens and leads to development of the cold SST_EEIO_ bias through enhanced upwelling in the coupled model. Results from a seasonal forecast experiment where the atmosphere is strongly relaxed towards atmospheric winds, temperature, and moisture from ERA5 (FC_AtmNudge_2hr) demonstrates that the cold thermocline bias present in ORAS5 impacts the surface only when coupled wind feedback is permitted.

Investigation of the state-dependence of atmospheric errors shows that warmer SSTs are associated with overly strong easterlies, even in uncoupled experiments, while cooler SSTs are associated with smaller wind biases. We note that SEAS5 forecasts develop a warm SST bias in the WEIO (Johnson et al. 2019) which likely further enhances the easterly bias in the coupled runs. Local precipitation response to SST_EEIO_ anomalies is underestimated when compared to reanalyses, with a stronger underestimation by the uncoupled compared to the coupled forecasts, which may explain the still too low wind sensitivity of uncoupled forecasts despite correct SSTs in the WEIO. Considering relationships of winds in the EEIO with zonal SST gradients along the equatorial Indian Ocean, we find better agreement of the forecasts with reanalyses, although easterlies are still too strong for high SSTs (i.e., strongly positive zonal SST gradients). For the coupled runs, the cause for the improved relationship is likely the removal of the effect of the warm WEIO bias by considering the zonal SST gradient. A potential cause for the remaining bias is too weak local diabatic heating and associated wind response in the EEIO arising from the underestimation of the local precipitation response, but remote errors such as the underestimated relationship between SST_EEIO_ and ENSO and its effect on atmospheric circulation likely play a role as well. For the uncoupled forecasts, overestimation of the anticyclonic circulation over the northern subtropical associated with the too strong southeast Asian monsoon may play an additional role.

Diagnostics of state-dependent relationships between subsurface ocean temperatures and SST_EEIO_ show that there are two distinct regimes: the warm regime with deep thermocline and weak sensitivity of SSTs to winds and a cold regime with a shallow thermocline and a strong sensitivity of SSTs to winds. Coupled forecasts predominantly reside in the cold regime (opposite to observations), in which SST errors amplify quickly in the presence of wind errors.

We conclude that there are two fundamental and independent sources of errors that lead to the SST errors in seasonal forecast. The first one is of atmospheric nature and is largely related with too stable easterly circulation present over the whole equatorial Indian Ocean, characterized by the lack of response of the local winds to local surface heating in the EEIO. This induces an easterly bias which leaves the model predominantly in a state with a shallow thermocline and cold SSTs in the EEIO. Circulation errors arising from precipitation biases in the southeast Asian monsoon regions play a role as well. Although SSTs and winds are clearly coupled two-ways, our results based on uncoupled forecasts and forecasts with a nudged atmosphere suggest that in the present case the biases can be largely attributed to a too weak response of the atmosphere to SST variations. The second error is of oceanic origin, associated with a too shallow thermocline present already in the initial conditions from ORAS5, which enhances the SST errors arising from errors in the wind. The markedness of this error varies with ocean initial conditions, which depend on both the quality of the assimilation and the ocean model. The version of the ocean model used for the forecast also plays a non-negligible role at seasonal time scales and can substantially amplify the subsurface errors in the initial conditions due to the strength of the atmosphere-ocean coupling in this region.

EEIO biases exhibit considerable low-frequency variations during the SEAS5 hindcast period 1981–2021, with largest cold SST bias during the 1993–2011 period. Furthermore, we found strong temporal correlation between ensemble mean forecasts of SST_EEIO_ and their error during 1981–2021. This suggests state-dependence of the forecast errors in the EEIO, but likely also of their remote impact. Future research could apply the here-presented diagnostics to longer hindcast data sets such as the coupled Seasonal Forecasts of the 20th century (Weisheimer et al. 2022), to elucidate the role of non-stationarity of the bias on decadal IOD forecast skill variations (such as those documented by Hu et al. 2023). To conclude, these results warrant further investigation and underline the need for more sophisticated and state-dependent forecast calibration.

### Electronic supplementary material

Below is the link to the electronic supplementary material.

Supplementary Material 1

## Data Availability

SEAS5 and ERA5 data are available from the Copernicus Climate Data Store. Other datasets analysed during the current study are not publicly available due to their experimental nature but are available from the corresponding author on reasonable request.
