# Supplementary material for: Assessment of seasonal forecasting errors of the ECMWF system in the eastern Indian Ocean
Source: Clim Dyn. 2023 Oct 12;62(2):1391–406. doi: 10.1007/s00382-023-06985-3 (PMC10827862; doi:10.1007/s00382-023-06985-3)
Supplement: Supplementary file 1 — Supplementary Material 1 [file 382_2023_6985_MOESM1_ESM.docx]

**Assessment of seasonal forecasting errors of the ECMWF system in the eastern Indian Ocean**

**Supplementary Material**

Michael Mayer^1,2, *^, Magdalena Alonso Balmaseda^1^, Stephanie Johnson^1^, Frederic Vitart^1^

^1^Research Department, European Centre for Medium-Range Weather Forecasts, Reading, UK

^2^Department of Meteorology and Geophysics, University of Vienna, Vienna, Austria

[^*^michael.mayer@ecmwf](mailto:*michael.mayer@ecmwf).int


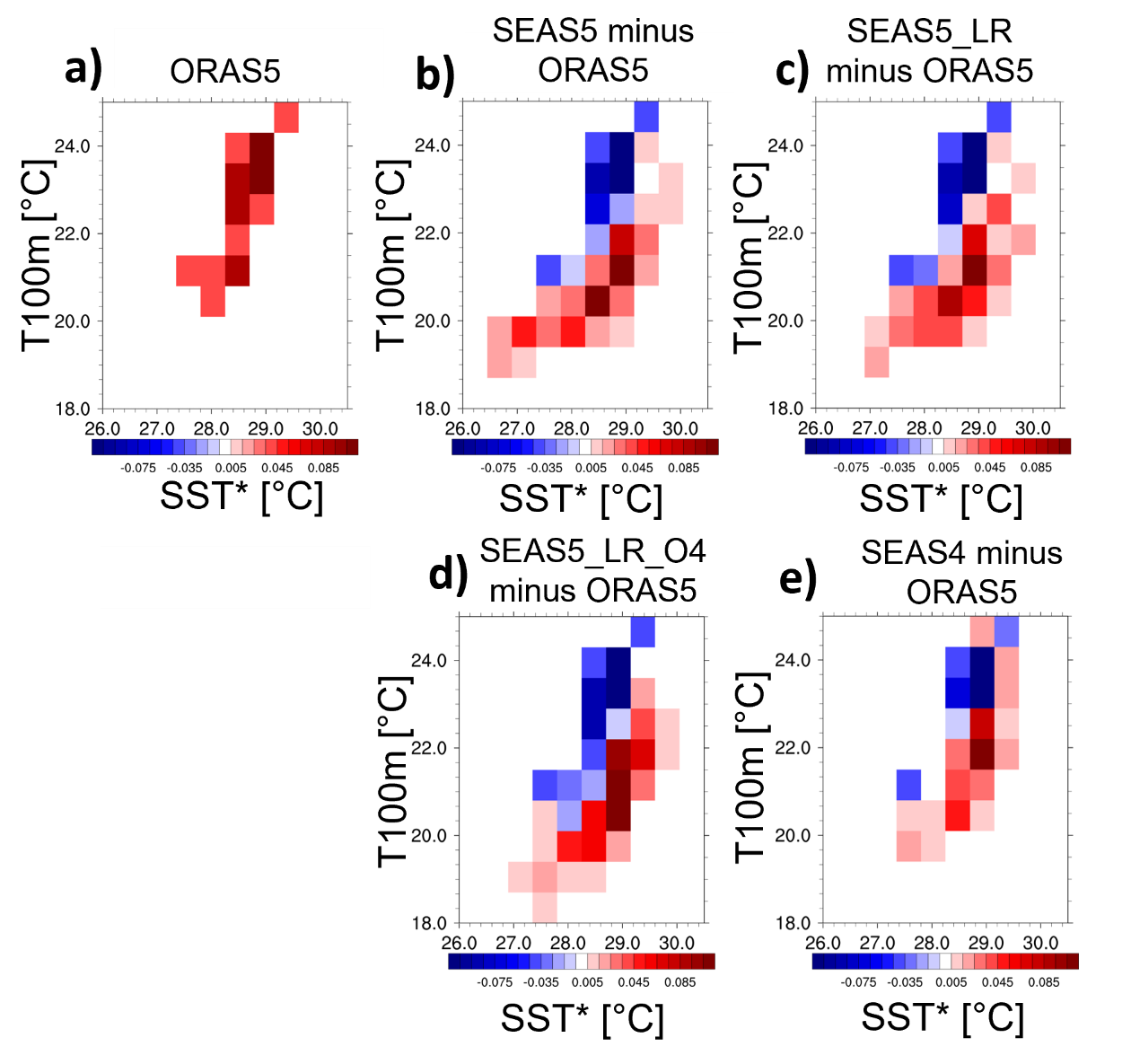


Figure S1. Heat maps showing joint frequency distribution of JJA SST* and T100 in the EEIO. Distributions shown in (a) are based on ORAS5. Other panels show differences in distributions between (b) SEAS5 hindcasts and ORAS5 (c) SEAS5_LR and ORAS5, (d) SEAS5_LR_O4 and ORAS5, and (e) SEAS4 and ORAS5. The ORAS5 distribution is based on 1993-2015 JJA data. Distributions of hindcasts are based on 10 members per start date (initialized on 1 May 1993-2015).


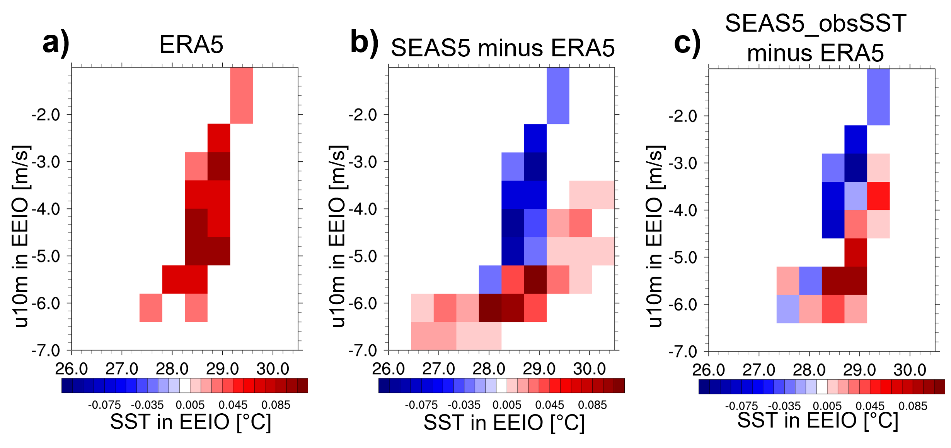


Figure S2. Heat maps showing joint frequency distribution of JJA SSTs in EEIO and 10m u-wind in JJA in EEIO. Distributions shown in (a) are based on ERA5. Other columns show differences in distributions between (b) SEAS5 hindcasts and ERA5, and (c) SEAS5_obsSST and ERA5. ERA5 distributions are based on 1993-2021 JJA data. Distributions of hindcasts are based on 10 members per start date (initialized on 1 May 1993-2021).


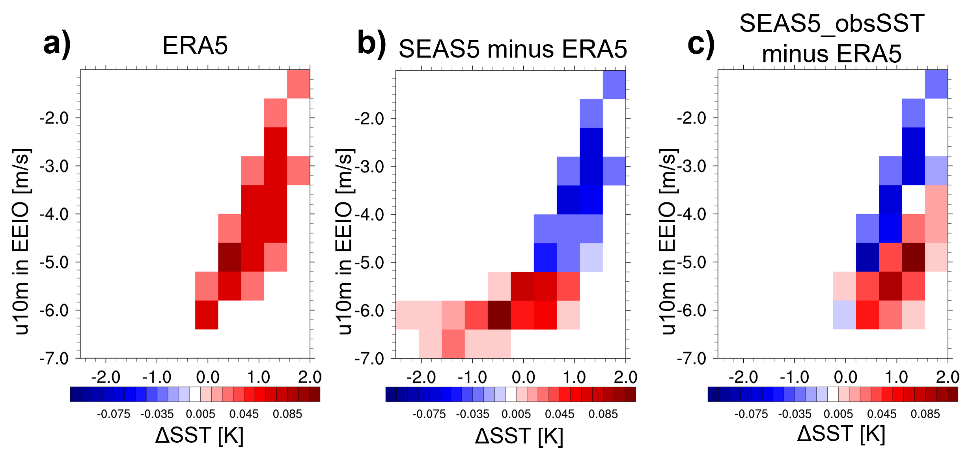


Figure S3. Heat maps showing joint frequency distribution of JJA west-east SST gradients in the Indian Ocean (ΔSST) and 10m u-wind in JJA in EEIO. Distributions shown in (a) are based on ERA5. Other panels show differences in distributions between (b) SEAS5 hindcasts and ERA5 (c) SEAS5_obsSST. ERA5 distribution is based on 1993-2021 JJA data. Distributions of hindcasts are based on 10 members per start date (initialized on 1 May 1993-2021).


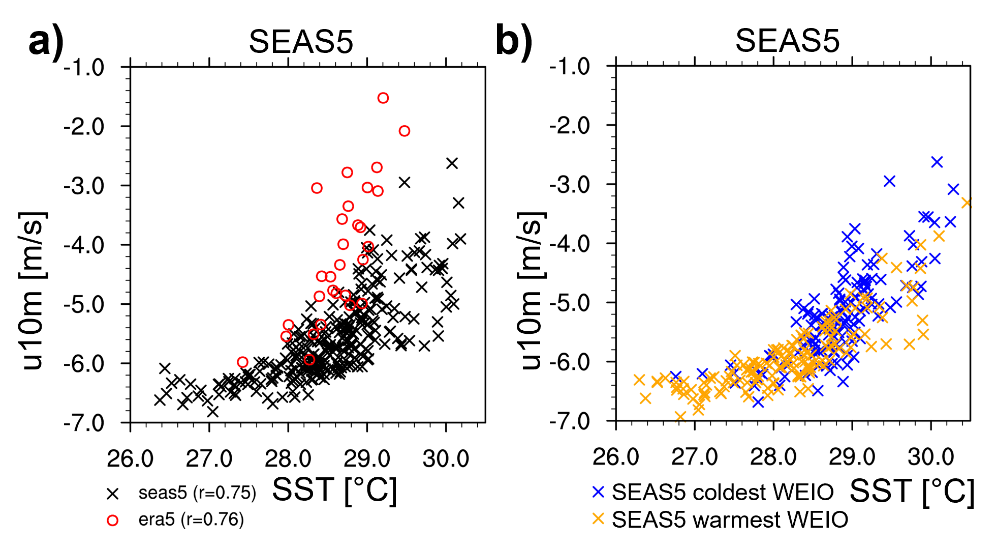


*Figure S4. a) Relationship between SST in EEIO and u10m in EEIO in JJA from SEAS5 (May start dates) and ERA5 (as Fig. 8a in the main text); b) as a), but taking 5 ensemble members of each forecast (selected from 25 member ensemble) with warmest and coldest SSTs in the WEIO, respectively*


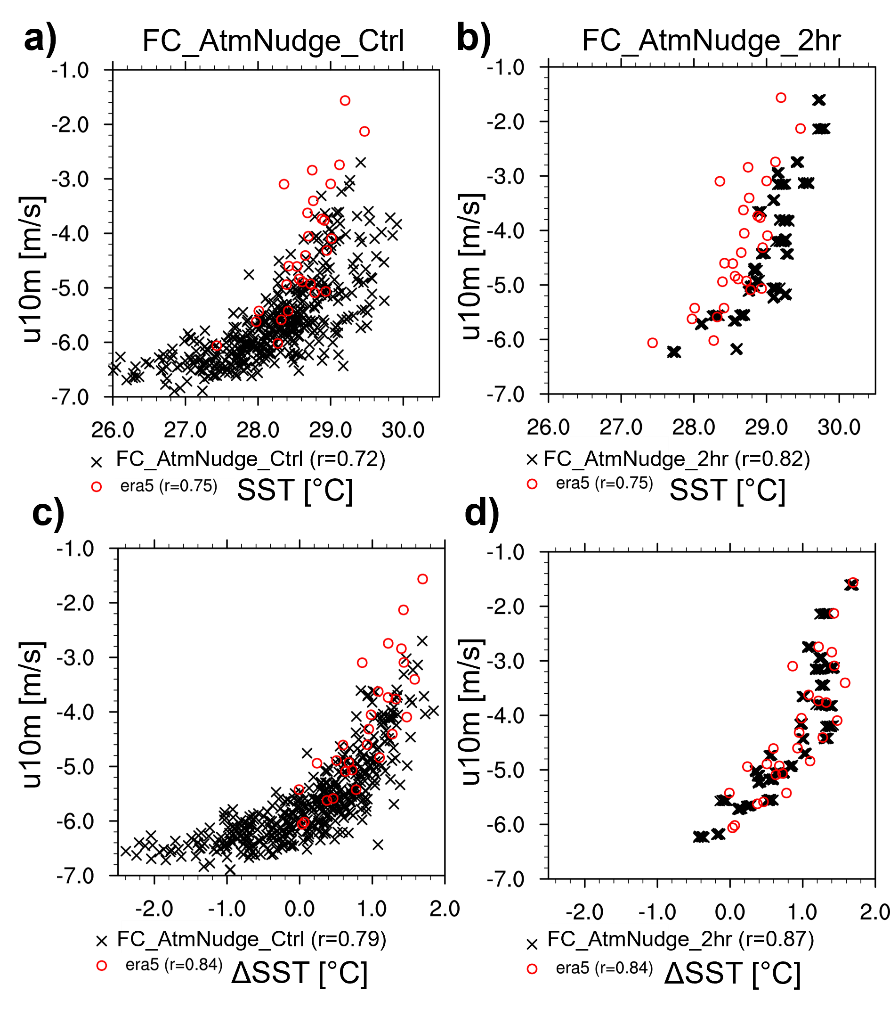


Figure S5. Scatter diagrams of (a,b) JJA SSTs in EEIO against 10m u-wind and (c,d) JJA west-east SST gradients against 10m u-wind in JJA in EEIO. Black crosses show single members (15 per start date) of (a,c) FC_AtmNudge_Ctrl and (b,d) FC_AtmNudge_2hr forecasts initialized on 1 May 1993-2020 and red circles relationships based on ERA5 1993-2020. FC_AtmNudge_Ctrl is shown to demonstrate the similarity with SEAS5 and cleanly assess the impact of the atmospheric nudging on the relationships.
